# Randomized controlled trial of intermittent hypoxia in Parkinson’s disease: study rationale and protocol

**DOI:** 10.1186/s12883-024-03702-3

**Published:** 2024-06-22

**Authors:** Jules M. Janssen Daalen, Marjan J. Meinders, Soania Mathur, Hieronymus W.H. van Hees, Philip N. Ainslie, Dick H.J. Thijssen, Bastiaan R. Bloem

**Affiliations:** 1 Radboud University Medical Center, Department of Neurology, Donders Institute for Brain, Cognition and Behavior, Center of Expertise for Parkinson & Movement Disorders, Nijmegen, The Netherlands; 2 Radboud University Medical Center, Department of Medical BioSciences, Nijmegen, The Netherlands; 3 UnshakeableMD, Oshawa, ON Canada; 4 Radboud University Medical Center, Department of Pulmonary Diseases, Nijmegen, The Netherlands; 5 University of British Columbia, Center for Heart, Lung and Vascular Health, School of Health and Exercise Sciences, Kelowna, Canada

**Keywords:** Parkinson's disease, Disease modification, Disease-modifying tria, Hypoxia, hypoxic conditioning, Mitochondrial dysfunction, Oxidative stress

## Abstract

**Background:**

Parkinson’s disease (PD) is a neurodegenerative disease for which no disease-modifying therapies exist. Preclinical and clinical evidence suggest that repeated exposure to intermittent hypoxia might have short- and long-term benefits in PD. In a previous exploratory phase I trial, we demonstrated that in-clinic intermittent hypoxia exposure is safe and feasible with short-term symptomatic effects on PD symptoms. The current study aims to explore the safety, tolerability, feasibility, and net symptomatic effects of a four-week intermittent hypoxia protocol, administered at home, in individuals with PD.

**Methods/Design:**

: This is a two-armed double-blinded randomized controlled trial involving 40 individuals with mild to moderate PD. Participants will receive 45 min of normobaric intermittent hypoxia (fraction of inspired oxygen 0.16 for 5 min interspersed with 5 min normoxia), 3 times a week for 4 weeks. Co-primary endpoints include nature and total number of adverse events, and a feasibility-tolerability questionnaire. Secondary endpoints include Movement Disorders Society-Unified Parkinson’s Disease Rating Scale (MDS-UPDRS) part II and III scores, gait tests and biomarkers indicative of hypoxic dose and neuroprotective pathway induction.

**Discussion:**

This trial builds on the previous phase I trial and aims to investigate the safety, tolerability, feasibility, and net symptomatic effects of intermittent hypoxia in individuals with PD. Additionally, the study aims to explore induction of relevant neuroprotective pathways as measured in plasma. The results of this trial could provide further insight into the potential of hypoxia-based therapy as a novel treatment approach for PD.

**Trial registration:**

ClinicalTrials.gov Identifier: NCT05948761 (registered June 20th, 2023).

## Introduction

Parkinson’s disease (PD) is a chronic and progressive neurodegenerative disorder for which only symptomatic treatments are currently available. The pathophysiology of PD is complex and involves multiple mechanisms, including mitochondrial dysfunction and oxidative stress. Although there have been significant advances in understanding the molecular and cellular mechanisms underlying PD, disease-modifying therapies that can halt or slow down the disease progression are lacking [1]. Therefore, there is an urgent need for novel therapeutic strategies that can provide symptomatic relief and neuroprotection.

Recent preclinical studies have suggested that moderate hypoxia, the condition of low oxygen availability, may offer a promising non-pharmacological treatment avenue in PD [2]. Hypoxia activates evolutionarily well-preserved adaptive mechanisms that enhance neuronal viability and survival [3, 4]. Several animal studies have reported neuroprotective effects of hypoxia in neurodegenerative and mitochondrial disease models [5–9]. One potential mechanism through which hypoxia treatment may improve PD is via the activation of the Hypoxia Inducible Factor 1 (HIF-1) pathway. HIF-1 is a transcription factor that plays a crucial role in cellular adaptation to hypoxia by regulating the expression of genes involved in energy metabolism, angiogenesis, and neuroprotection [10–12]. Short-term activation of HIF-1 may lead to increased nigrostriatal dopaminergic activity [13–16], while long-term repeated exposure to moderate hypoxia may improve the systemic tolerance of cells and tissues to subsequent more severe stimuli, a process called hypoxic conditioning [2, 11, 17]. Interestingly, HIF-1 is necessary for the disease-modifying effect of exercise in preclinical substantia nigra models [18]. Intermittent hypoxia - the interspersion of hypoxia with brief periods of normoxia – therefore seems a promising method of administration as it allows for recovery and prevents tolerance to the hypoxic trigger [2, 19]. Several intermittent hypoxia studies have been conducted in elderly and fragile populations [20–25], and in a previous phase I study, we have demonstrated the safety and feasibility of hypoxia trials in individuals with mild to moderately severe PD (manuscript in preparation).

In this phase 1b-2a randomized controlled trial, we aim to investigate the safety, tolerability, feasibility, and net symptomatic effects of a four-week intermittent hypoxia protocol at participants’ homes. Mechanistic effects are explored by investigating putative neuroprotective blood markers. We hypothesize that a remote intermittent hypoxia protocol in individuals with Parkinson’s disease is (1) safe and feasible in individuals with PD without cardiorespiratory comorbidity and that a 4-week protocol (2) improves symptomatic and functional outcomes, and (3) increases hemoglobin, hematocrit and induces markers of neuroprotection.

## Study objectives

### Primary objective

(i) to explore the safety (number and nature of AEs) and feasibility and tolerability (questionnaire) of remote administration of intermittent hypoxia interventions in PD.

### Secondary objectives

(ii) to explore the net effects of a 4-week intermittent hypoxia protocol on a battery of standardized and functional scales, including Movement Disorders Society-Unified Parkinson’s Disease Rating Scale parts II and III.
(iii) to assess the net effects of a 4-week intermittent hypoxia protocol on plasma biomarkers, indicative of neuroprotective pathway induction.

### Methods/Design

#### Design

We will study the safety and net effects of a 4-week intermittent hypoxia protocol in a two-armed randomized controlled trial. The design and content of several elements of the current study is based on a previous exploratory phase I multiple N-of-1 trial [26]. 

### Population

We will include 40 individuals with PD, clinically confirmed by a movement disorders-specialized neurologist.

**Inclusion criteria**.

- Able and willing to provide written informed consent.
- Clinical diagnosis of Parkinson’s disease by a movement disorders-specialized neurologist.
- Hoehn and Yahr stage 1 up to and including 3.

### Exclusion criteria

An individual who meets any of the following criteria will be excluded from participation in this study:

- Individuals with a diagnosis of restrictive and obstructive pulmonary diseases, cardiac output deficits, sleep apnea (or signs or symptoms thereof), excessive alcoholic intake, congestive heart failure and coronary artery disease NYHA classes III and IV.
- Arterial blood gas abnormalities at screening procedure (see limits and stop criteria in *Supplementary Materials*).
- Individuals with shortness of breath or other airway or breathing-related inconvenience related to lack of dopaminergic medication.
- Unable to come to the clinic for measurements in OFF phase, or having unstable dopaminergic medication.
- Individuals with active deep brain stimulation (DBS).

#### Sample size calculation

As this involves a phase 1b-2a trial with no previous comparable insights to base a calculation upon, formal sample size calculations will not be possible. Similar to our previous hypoxia trial [26], *n* = 20 per treatment arm is considered sufficient to assess nature and number adverse events, as well as the feasibility (Appendix III) of performing a hypoxia trial remotely.

#### Recruitment and consent

Individuals with PD will be recruited from a national recruitment website for PD-related research (*ParkinsonNEXT.nl*). Participants will receive an e-mail with detailed information on the study, including an overview of time investment and risks of participation. Individuals will have a consultation by telephone with the study contact person to answer questions about the research after receiving the information. If the participant is still interested, a screening questionnaire will be sent before the physical screening procedure. The coordinating investigator will contact the participant before in-clinic screening procedure if potential exclusion criteria are met, to prevent any unnecessary participant visits. Before the on-site screening begins, the informed consent form is signed.

### Premature termination of participants

Participants have the right to withdraw consent at any moment during the study period without any consequences. After withdrawal, subjects will not be replaced during the study. Follow-up of withdrawn participants will be conducted by telephone consultation within one week after the last intervention. The occurrence of adverse events will be evaluated actively on a weekly basis. If permitted by the participant, the reason for study withdrawal will be collected.

### Premature termination of the study

Informed by our phase I study, we do not expect to terminate this study on the basis of adverse events. However, the study will be terminated or be put on hold if the intervention appears to be harmful or if serious adverse events occur probably or definitely due to trial interventions. Any issue regarding study continuation will be discussed in close consultation with the Data Safety Monitoring Board (discussed hereafter).

### Interventional medical device

A commercially available hypoxic generator (*b-Cat ALT-120*, B-cat High Altitude, Tiel, the Netherlands) is used, which is similar to devices regularly used in hypoxic training studies [26–38]. The intervention will be primarily administered at the participant’s home. Therefore, the generator is modified in collaboration with the manufacturer, such that the device automatically titrates the correct fraction of inspired oxygen (F_I_O_2_) of 0.16 in a closed feedback loop with an F_I_O_2_ sensor, and automatically regulates the intervention per protocol (five times five minutes hypoxia interspersed with five minutes of normoxia, totaling 45 min). This protocol was selected based on (1) the balance between safety and clinical effects as derived from our first study [26] (manuscript submitted), (2) the literature on the spectrum of dose-related effects of intermittent hypoxia [19], and (3) the multidimensional respiratory dysfunction in PD in response to (severe) hypoxia observed in our previous study (manuscript submitted), limiting the intensity of the hypoxic stimulus as per our current stop criteria (*Appendix II*).

The participant wears a pulse oximeter that feeds back to a computer linked to the generator. When SpO_2_ drops below 80% or SpO2 input is lost for one minute, the device automatically switches off and defaults to room air. As an extra safety measure, a safety button needs to be pressed every couple of minutes in response to a flashing light signal by the participant during any intervention. When the button is not pressed, the device switches off and the participant breathes room air. Lastly, a continuous output oxygen measurement system verifies appropriate dosing. Other device settings that are normally customizable are now disabled or moved to the inside of the machine and can therefore not be reached or modified by the participant. This will increase the safety of using the hypoxicator in the home environment. Participants receive elaborate on-site instructions and a tailored instruction manual for own use. A technical schematic of the device and its safety modifications is added in *Appendix IV*.

### Outcomes and measurements

#### Primary outcomes: safety and feasibility

This is the first trial that investigates the deployment of an intermittent hypoxia protocol in a remote setting. In our previous trial, we tested single sessions of different hypoxia training protocols in PD (manuscript submitted). Building on that experience, the safety, tolerability and feasibility of a month-long intermittent hypoxia protocol in PD are the primary outcomes of this study (Table 1).

**Table 1:** Co-primary and secondary outcome measures

Co-primary study outcomes	Remarks
Adverse events	Number and nature
Feasibility and tolerability questionnaire (*Appendix III*)	Categorized as per a widely used feasibility framework [49]. Items in each category (e.g. acceptability, expectancy) were subsequently inspired by a variety of healthcare feasibility questionnaires.
**Secondary study outcomes**	
Movement Disorder Society-Unified Parkinson’s Disease Rating Scale (MDS-UPDRS) part II and part III score	Experiences limitations in activities of Daily Living (part II, participant-reported) and Examination of motor symptoms (part III, clinician-assessed)
Purdue pegboard test (PPT)	Left hand, right hand, both hands
Timed Up & Go Test (TUGT)	Time and number of steps, average of two trials
Six-minute walking test (6MWT)	Distance covered
Parkinson’s disease questionnaire-39 (PDQ-39)	Self-completed
Accelerometry-measured resting tremor and pronation-supination	Movisens® Move 4 activity sensor
Plasma markers of target engagement and blood biomarkers	Hemoglobin, neurofilament light chain (NfL), clusterin, GFAP, UCH-L1, BDNF (brain-derived neurotrophic factor)

### Secondary outcomes: functional and motor tests

The selection of motor and functional outcome measures is based on the findings of our previous hypoxia trial (manuscript in preparation) and aims to measure for the first time the net symptomatic effects of a one-month intermittent hypoxia protocol in PD [26]. To the existing motor test battery, we added the MDS-UPDRS part II and 6-minute walking test (Table 1) to better account for functional effects, including exercise capacity.

### Secondary outcomes: plasma biomarkers

Plasma biomarkers are included as hypothesis-generating outcome measures and are based on their value in target engagement (hemoglobin, hematocrit) and sensitivity to either exercise and longitudinal disease progression.

Neurofilament-light chain (NfL) is associated with motor decline in PD [39, 40] and is reduced by exercise [41]. Clusterin is a potential important regulator of exercise effects, and might positively influence neuro-inflammation and be inversely associated with cognitive function [42–45]. Glial fibrillary acidic protein (GFAP) and ubiquitin carboxy-terminal hydrolase L1 (UCH-L1) are acute biomarkers of brain injury and might therefore give influence in whether this is upregulated in response to hypoxia as a stressor [46]. Brain-derived neurotrophic factor (BDNF) is potentially upregulated by hypoxia and exercise [47, 48]. As research into PD plasma biomarkers keeps accelerating, novel insights will be used to modify this preliminary biomarker selection. Extra plasma will be stored for additional analyses where appropriate. Any necessary protocol amendments will be coordinated with the medical-ethical committee.

### Other study parameters

In addition to the outcome measures, information about age, gender, disease severity (Hoehn and Yahr scale), disease duration, levodopa equivalent daily dose (LEDD) and use of concomitant medication (including any changes during trial participation) are collected.

### Measurement frequency

In-clinic measurements will be performed at baseline, in the week after the 4-week intervention period and 4 weeks after the last intervention session, except for the feasibility questionnaire that will be sent once, immediately after the intervention period. Adverse events are evaluated on a weekly basis.

### Randomization, blinding and treatment allocation

The study is participant- and outcome assessor-blinded. Allocation to intervention order will be randomized by a computer-generated randomization scheme in *R* using block randomization. The lab technician is not blinded for safety and monitoring purposes and will oversee the randomization process. Although ventilation is expected to increase with ~ 20% mostly due to increased tidal volume and possibly respiratory rate, the extensive evidence-based experience of the study team and study advisors is that participants will not notice changes in their breathing at an F_I_O_2_ of 0.16 [50–53]. Indeed, blinding was assessed and successful in our latest hypoxia trial in PD where participants were exposed to multiple independent interventions of continuous and intermittent mild to moderate hypoxia (manuscript submitted).

### Study procedures

#### Screening procedure

First, participants will fill in an online questionnaire (Appendix I) about cardiac or pulmonary diagnoses or symptoms and smoking, to exclude individuals with relevant cardiorespiratory abnormalities. If this questionnaire does not yield any exclusion criteria, participants are invited for an in-hospital screening procedure.

During this screening procedure, informed consent will first be ascertained, and the informed consent form will be signed. Subsequently, participants will be screened for cardiac abnormalities using an electrocardiogram and blood for baseline measurement is drawn and processed per standard plasma procedures. Then, participants undergo neurological testing, and finally participants will be blindly exposed to the active study intervention under arterial blood gas (ABG) and cardiopulmonary monitoring of an experienced pulmonary lab technician. ABG will be taken at the start of the intervention, and in the last 5 min of hypoxia. If oxygen saturation reaches 82%, an extra ABG will be taken in-between to investigate arterial pO_2_. Stop criteria during this intervention are pre-defined (Appendix II). Continuous non-invasive cardiorespiratory monitoring is conducted using the Cosmed® metabolic monitoring system (*Quark* metabolic cart for cardiopulmonary exercise testing [CPET], *COSMED Srl*, The Metabolic Company, Italy).

#### Intervention procedures

Interventions will be conducted three times a week, for four weeks in total. The first intervention will take place in the hospital under supervision of a pulmonary technician. Any subsequent intervention will take place at home, with a pulmonary technician present for instructions and monitoring at least once. If necessary for safety purposes, this technician will be additionally present for one or more subsequent interventions. After that, remote expert assistance is present through a live (video)call during each session in the first intervention week. Supervision can be extended when necessary for safety during subsequent weeks. Mandatory weekly videocalls ascertain adequacy of administration, safety and compliance. Participants are explicitly encouraged to spread the three interventions over any week as much as possible. By exception, an occasional intervention on the day following a previous intervention will be allowed.

Before the first session, the pulmonary technician will explain the working mechanism of the machine and will give ample time for questions. This includes the auto-off function of the machine, and when to contact the study team. When one individual has completed the Intervention phase, the machine will be collected and will be delivered to the next participant. In-between, machine parts that are part of the breathing circuitry will be cleaned for re-use.

### Outcome measurement

Participants will be tested in-clinic for all secondary outcome measures except PDQ-39 in a practically defined OFF-medication state (> 12 h after intake after the last dose of dopaminergic medication). This OFF testing will be conducted in the morning and is a widely used and proportional method of ascertaining no relevant effects of exogenic dopamine on outcome measurement [54–58]. PDQ-39 is filled in by participants through an online survey. Because driving capability may be influenced by being OFF, we ask participants to refrain from driving to the in-hospital appointments themselves. Instead, they may be driven by partner, family, acquaintance or public transport. Travel expenses for clinic visits are covered.

### Statistical analysis

#### Primary study outcomes

Safety (i.e., number and nature of adverse events) and the feasibility (i.e., questionnaire (details)) will be analyzed using descriptive statistics.

#### Secondary study outcomes

Secondary study outcomes (functional and motor testing, and plasma markers of target engagement) will be analyzed by linear mixed models (for linear effects between T0, T1 and T2), and if applicable, with post-hoc analyses using ANOVA.

### Interim analysis

An interim analysis of study progress and safety will be performed after 20 individuals have completed the study. This will not involve a futility analysis because of the early phase of this clinical trial.

### Monitoring and registration

#### Quality assurance and monitoring

The study will be monitored regarding the health, safety and rights of participants, protocol adherence and quality of data and data reporting during this trial at study initiation, twice during the study (on-site) and once at study completion. On-site monitoring visits (performed by a specialized study monitor) will assess the progress of the study, study procedures, used study materials and identify any concerns that result from review of the subject Informed Consent documentation, study records, collected data and study management documents. The study monitor will also ensure the Investigator adheres to all applicable regulations.

### Data safety monitoring board

A data safety monitoring board (DSMB) will be established, which will consist of a PD-specialized neurologist, an anesthesiologist, a pulmonologist, an epidemiologist, and a patient representative. The DSMB will monitor the occurrence of adverse events and the study-related overall health of participants in this trial. An interim analysis will be performed after the successful completion of the study procedures by the first twenty participants to provide the DSMB with the latest recruitment data and safety outcomes, after which the Board will come to a consensus regarding study continuation or any adjustments. No member of the board has a conflict of interest with the sponsor of the study.

## Discussion

In this phase 1b-2a randomized controlled trial, we will for the first time assess the safety and net effects of a four-week intermittent hypoxia protocol in people with mild to moderate PD.

Various previous studies have investigated hypoxia in PD. These trials included both healthy adults (with or without intense exercise interventions in hypoxic conditions [35, 59, 60]) as well as individuals with cardiovascular or pulmonary disease [20–22, 61, 62]. As hypoxia protocols differ importantly between studies, we have weighed the evidence and created a balance between safety and efficacy [19]. In this, we specifically consider the respiratory dysfunction in people of older age, and even more in people with PD, including a reduced hypoxic ventilatory response [63–65]. Therefore, we deploy a slightly higher F_I_O_2_ than deployed in some other hypoxic training studies, although the proposed protocol is still regarded a potent stimulus [19]. 

Previous studies found increases in hematopoiesis and cardiovascular fitness for comparable protocols, assuring target engagement [31, 66, 67]. These previously established beneficial effects on cardiovascular function in healthy humans are the prime reason to include the 6MWT as a marker of light- to moderate-intensity endurance [68]. Further exploration into the mechanisms of action is a specific objective of this study, and therefore, a selection of putative plasma markers of neuroprotection and neuronal injury is included in this study. It should be noted that for various of these markers, sensitivity to hypoxia and post-exposure timeline are unknown.

The HIF-1 pathway, which is regarded the prime mediating pathway for adaptive responses to hypoxic training protocols, is increasingly regarded as a potential target for the development of small-molecule approaches that can activate neuroprotective pathways in PD [69]. Various studies have been conducted with HIF enhancers or inhibitors of prolyl hydroxylase, the enzyme that constitutively breaks down HIF in normoxic environments. These studies have demonstrated neuroprotection in basal ganglia in parkinsonian models [3, 15, 70, 71], primarily through improving mitochondrial biogenesis, turnover and electron transport chain efficiency, and subsequently decreasing oxidative stress and pro-apoptotic pathway induction [72–74]. This clinical study may therefore additionally accelerate the translation of such preclinical insights into a target for repurposed pharmacological compounds [69]. It should be noted that the hypoxia response pathway is largely, but not completely, HIF-dependent [75], and that HIF-1 activation in normoxic conditions is not disease-modifying in some preclinical models of neurodegeneration [6, 7]. 

A prime novelty of this study is the remote, home-based nature of administration of a hypoxic training protocol. To the best of our knowledge, this approach has not been performed previously in individuals with a neurodegenerative condition, although remotely administered hypoxic protocols have been proposed previously [76]. Important considerations for the home-based use of an hypoxic intervention include the feasibility of intermittent hypoxia as a non-pharmacological intervention. Although in-center protocols have also pursued multiple-week protocols, home-based administration is a necessity for feasible application of low-burden intermittent hypoxia protocols in long-term practice. The same argument holds for the scalability of the intervention, as well as the long-term compliance to interventions in PD [77]. 

Some limitations of this study need to be considered. First, the intervention duration will likely be insufficient for structural or long-term symptomatic effects beyond the study period. Most previous studies that have investigated intermittent hypoxia in other populations have either not included long-term follow-ups or have not been able to show lingering effects beyond the intervention period [62, 78]. In this study, we therefore also included a longer-term follow-up of one month after the intervention period to assess the longer-term stability of effects on clinical symptoms and blood-based markers. Recent evidence suggests that interspersion of hypoxia with either hyperoxia or hypercapnia might respectively enable a more rapid recovery after hypoxia or enhance the potency of effect on mitochondrial metabolism, providing an opportunity for comparison to the current protocol [20, 79, 80]. This study includes a relatively low number of participants, which hampers its power to detect smaller treatment effects on several included standardized scales. Furthermore, this study design is unable to provide meaningful insight into the potential neuroprotective merits of intermittent hypoxia in PD, apart from yielding hypothesis-generating insights that may be derived from the plasma biomarker selection. Indeed, previous neurological biomarker studies in hypoxic training studies are limited to BDNF [81–85], demonstrating conflicting results. This highlights that despite the broad biomarker selection, biomarkers may only provide limited insight into the potential neuroprotective benefit.

In conclusion, intermittent hypoxia may represent a relatively simple, low-cost, and easily applicable and potentially scalable strategy that could be added to the current arsenal of non-pharmacological therapies for PD. This study is designed to investigate the safety and symptomatic effects of a multiple-week intermittent hypoxia protocol in PD and will thus provide essential insights for future studies to better understand its therapeutic potential in PD.

## Appendix I

1. Have you experienced chest pain in the past 5 years?
2. Have you fainted for an unexplained reason in the past 5 years?
3. Are you short of breath or exhausted after short or mild exertion, such as climbing the stairs or running errands?
4. Do you have heart failure or other heart disease?
5. Do you become stuffy or short of breath when lying down (in bed)?
6. Do you have (have you had) a heart murmur?
7. Do any of your parents, brothers, sisters or your children under the age of 50 have heart disease (myocardial infarction, arrhythmia, hypertrophy, cardiomyopathy, pulmonary QT syndrome or dysrhythmia)?
8. Did any of your parents, brothers, sisters or children die suddently/unexpectedly (sudden cardiac death)?
9. Do you have high blood pressure requiring multiple medications?
10. Do you smoke or have you smoked? No / Yes, namely: .
11. Have you lived or worked in an environment where there was a lot of smoking or you breathed other polluted air? No / Yes, namely: .
12. Do seasons, weather or air quality change your breathing to the extent that you may become short of breath or exhausted as a result?
13. Do you have any lung disease (e.g. asthma, bronchitis/COPD, pulmonary fibrosis, interstitial lung disease, emphysema)? If yes (which disease, severity, medication).
14. Do you often suffer from coughing or have a lot of trouble coughing up mucus?
15. Do you have sleep apnoea (breathing stops during sleep)?
16. On average, do you consume more alcoholic drinks than 3 units (men) or 2 units (women) per day?
17. Does not taking your parkinsonian medication make you feel stuffy, short of breath or exhausted, or does not taking it cause you to wheeze or whoop?
18. Do your parkinsonian symptoms become unbearable by delaying or not taking your morning medication?
19. Has anything changed about your parkinsonian medication (different drug or dose) in the past month?
20. Do you think you will need more or different parkinsonian medication in the next month?
21. Do you have any treatments that you find difficult(er) to momentarily interrupt or should not interrupt (e.g. as per your neurologist’s strict instructions), such as medication patches, deep brain stimulation (DBS) or a medication pump?

## Appendix II

### Stop criteria

Subjective parameters

- Dizziness, discomfort and stress scores higher than 7 on a 10-point Likert scale.

Vital parameters

- Heartrate > 140/min.
- Breathing frequency > 25 per minute.
- Oxygen saturation < 80%.

Arterial blood gas (during screening procedures)

- pO2 < 40 mmHg (5.33 kPa).
- pCO2 < 25 mmHg (3.33 kPa).
- pH > 7.55.

Other

- Occurrence of any serious adverse event, or the necessity to intervene for participant wellbeing.

## Appendix III

1. Feasibility questionnaire.
2. The interventions were uncomfortable.
3. Breathing through the mask was hard.
4. Breathing through the mask was frightening.
5. I become claustrophobic with the mask on my face.
6. The intervention was tiring.
7. The intervention was tedious to undergo.
8. The intervention was painful.
9. The intervention took too long.
10. I got a headache during interventions.
11. I felt short of breath during intervention.
12. I felt nauseous during the intervention.
13. I could undergo this intervention for longer periods.
14. I felt safe undergoing the intervention at home.
15. I would undergo this intervention at home from now on only with someone from the hospital present.
16. I would recommend this intervention to others with Parkinson’s disease.
17. I find this intervention logical as a Parkinson’s treatment.

## Appendix IV

**Figure:**
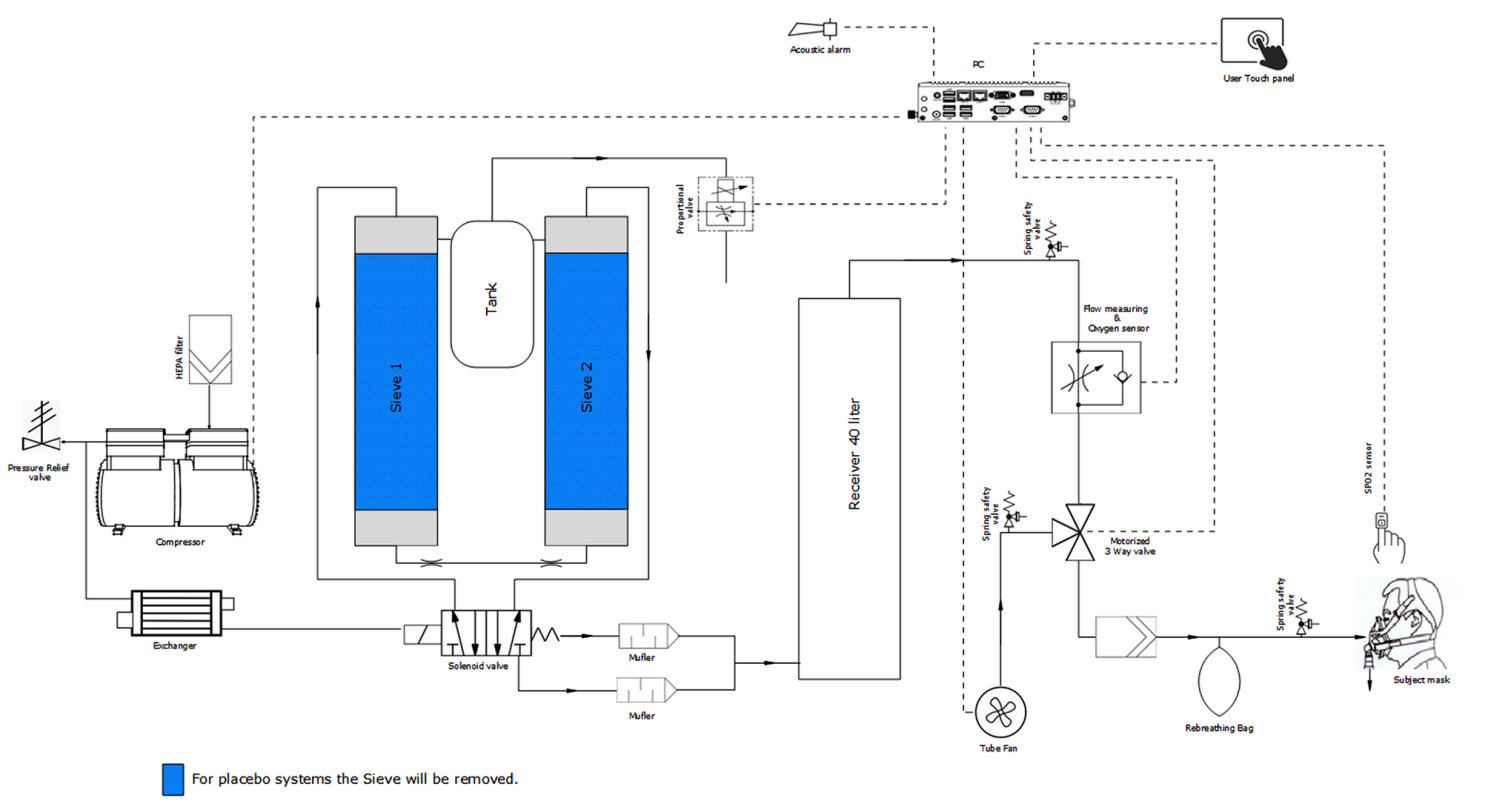
*

## Data Availability

Anonymized data will be shared with The Michael J. Fox Foundation for Parkinson’s Research (the study funder). This data may be kept for storage at a central repository either hosted by The Michael J. Fox Foundation, its collaborators, or consultants and will be kept indefinitely. Anonymized data will be made publicly available by the Foundation for the intended use of research in Parkinson?s disease as well as other biomedical research studies that may not be related to Parkinson?s disease

## Abbreviations

PD: Parkinson’s disease
HIF-1: Hypoxia-inducible factor 1
F_I_O_2_: Fraction of inspired oxygen
MDS-UPDRS: Movement Disorders Society Unified Parkinson’s Disease Rating scale
TUGT: Timed Up and Go Test
6MWT: Six-minute walking test
DSMB: Data safety monitoring board
ECG: Electrocardiogram
ABG: Arterial blood gas
LEDD: Levodopa-equivalent daily dose
BBB: Blood–brain barrier
MJFF: Michael J. Fox Foundation for Parkinson’s Research
